# Ultrasensitive Mach-Zehnder Interferometric Temperature Sensor Based on Liquid-Filled D-Shaped Fiber Cavity

**DOI:** 10.3390/s18041239

**Published:** 2018-04-17

**Authors:** Hui Zhang, Shecheng Gao, Yunhan Luo, Zhenshi Chen, Songsong Xiong, Lei Wan, Xincheng Huang, Bingsen Huang, Yuanhua Feng, Miao He, Weiping Liu, Zhe Chen, Zhaohui Li

**Affiliations:** 1 School of Physics and Optoelectronic Engineering, Guangdong University of Technology, Guangzhou 510006, China; zh999em@163.com; 2 Department of Electronic Engineering, College of Information Science and Technology, Jinan University, Guangzhou 510632, China; wallen-0407@163.com (L.W.); xchuang83@163.com (X.H.); huangbingsen@stu2016.jnu.edu.cn (B.H.); favinfeng@163.com (Y.F.); wpl@jnu.edu.cn (W.L.); 3 Guangdong Provincial Key Laboratory of Optical Fiber Sensing and Communications, Jinan University, Guangzhou 510632, China; yunhanluo@163.com (Y.L.); thzhechen@163.com (Z.C.); 4 Institute of Photonics Technology, Jinan University, Guangzhou 510632, China; zhenshichan@gmail.com; 5 State Key Laboratory of Optoelectronic Materials and Technologies and School of Electronics and Information Technology, Sun Yat-sen University, Guangzhou 510275, China; xiongsong1216@126.com (S.X.); tlzh88@jnu.edu.cn (Z.L.)

**Keywords:** micro-optical devices, fiber optics sensors, thermal effects

## Abstract

A liquid-filled D-shaped fiber (DF) cavity serving as an in-fiber Mach–Zehnder interferometer (MZI) has been proposed and experimentally demonstrated for temperature sensing with ultrahigh sensitivity. The miniature MZI is constructed by splicing a segment of DF between two single-mode fibers (SMFs) to form a microcavity (MC) for filling and replacement of various refractive index (RI) liquids. By adjusting the effective RI difference between the DF and MC (the two interference arms), experimental and calculated results indicate that the interference spectra show different degrees of temperature dependence. As the effective RI of the liquid-filled MC approaches that of the DF, temperature sensitivity up to −84.72 nm/°C with a linear correlation coefficient of 0.9953 has been experimentally achieved for a device with the MC length of 456 μm, filled with liquid RI of 1.482. Apart from ultrahigh sensitivity, the proposed MCMZI device possesses additional advantages of its miniature size and simple configuration; these features make it promising and competitive in various temperature sensing applications, such as consumer electronics, biological treatments, and medical diagnosis.

## 1. Introduction

Recently, owing to their lightweight, compact size, high sensitivity, and fast response, optical fiber-based sensors for temperature measurement have been extensively studied and applied in scientific research and different industrial areas [1]. Compared with traditional electric sensors, fiber optic sensors offer the distinguished features of immunity to electromagnetic interference, capability of distributed remote measurement, and durability against harsh environments such as high temperature and high pressure [2,3]. Based on these advantages, much more efforts have been made to exploit varieties of configurations and fabrication techniques for fiber sensors, such as D-shaped polarization-maintaining fiber loop mirror (DPM-FLM) [4], Fabry–Perot (FP) cavity formed by a hollow-core silica tube [5], long-period fiber gratings (LPFGs) [6], Michelson interferometer (MI) realized by a peanut-shape structure fiber [7], an liquid-sealed optical microfiber taper (OMT) [8], Mach–Zehnder interferometer (MZI) based on a microstructured optical fiber [9], a core-offset MZI based on a non-zero dispersion-shifted fiber (NZ-DSF) [10]. Among numerous fiber sensor schemes, MZI-based fiber sensors are always chosen for temperature, strain, torsion, and refractive index sensing, because of their high sensitivity to environmental variations [11,12,13]. An in-fiber MZI structure was reported as a wavelength selective filter to enhance the temperature sensing capability for an all-fiber temperature sensor [14].

Most of in-fiber MZI sensors utilize some mode-field-mismatched structures, such as micro-air-holes [15], and waist-enlarged fiber bitapers [16], to excite different cladding modes which are intended to interfere with the core mode. However, both cladding modes and core mode participate in the interference propagate in silica, thus they have similar mode effective refractive indicies (RI), which results in a sensing area of up to a few centimeters long, in order to accumulate enough phase difference [15]. Moreover, for a temperature sensor, large thermal coefficient of the sensing area material is of great benefit to the sensitivity improvement. In contrast, the thermal expansion and thermo-optic coefficients (TOCs) of silica-based optical fibers are small, which limits the sensitivities of the reported MZI-based fiber temperature sensors to hundreds even tens of picometers per centigrade [15,16]. Recently, some research has sought to improve the temperature sensitivity with the assistance of high TOC liquids. For instance, a high TOC liquid modified photonic crystal fibers (PCFs)-based multimode interferometer (MMI) temperature sensor was demonstrated with a high sensitivity of ~14.72 nm/°C [17]. By selectively filling high TOC liquids into one and two air holes of PCF innermost layer, the temperature sensitivities of the in-fiber MZI sensors have been enhanced to 16.49 nm/°C [18] and ~7.3 nm/°C [19] respectively. However, these liquid-modified PCF-based MZIs not only possess complicated configurations and high fabrication requirements, but also still have sensing area lengths of several centimeters.

In this work, we propose and demonstrate a simple, miniature, and ultrasensitive temperature sensor based on a liquid-filled in-fiber MZI. The MZI structure is fabricated by embedding a D-shaped fiber (DF) between two SMFs, to form a half-side-opened microcavity (MC) by use of fiber fusion splice. Due to the large effective RI difference between the DF and MC, the scale of MCMZI can be reduced to several hundred even tens of micrometers. Based on the MZI structural characteristics, a wide range of temperature sensitivities from 33.66 nm/°C to −84.72 nm/°C have been experimentally achieved by filling different RI liquids with high TOC into the MC. Experimental results show that the proposed MCMZI sensor with 456μm-length cavity filled with a liquid of RI ~1.482 has an ultrahigh temperature sensitivity of −84.72 nm/°C, which represents the highest for the in-fiber sensors reported so far, to the best of our knowledge. The simple fabrication process, miniature size, cost effectiveness, and ultrahigh sensitivity make it a competitive fiber sensor in highly sensitive temperature applications.

## 2. Structure Principles and Fabrication Methods

**Figure 1:**
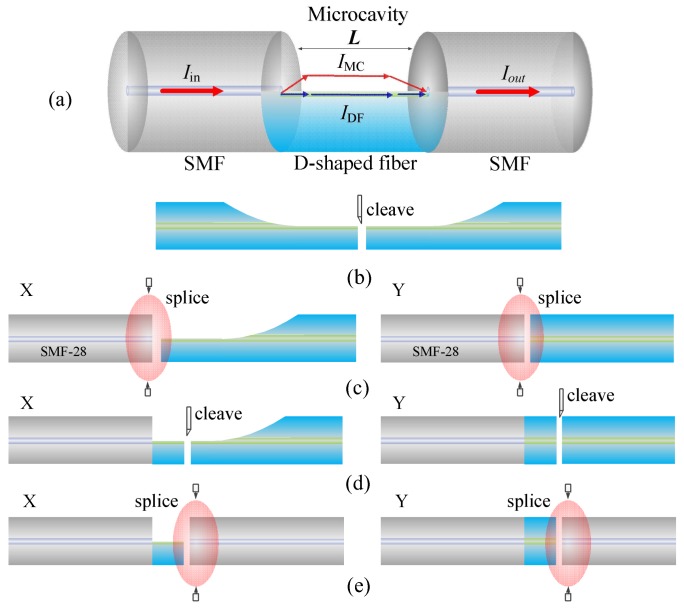
(**a**) Schematic diagram for the proposed MCMZI sensor; (**b**–**e**) Device fabrication procedures.

The schematic diagram of the proposed MCMZI is shown in Figure 1a, which is based on a segment of DF sandwiched between two SMFs. The side-opened MC above the DF allows reversible replacement of various RI liquids. The two special splicing joints at the front and rear ends of MC work as the beam splitter and combiner respectively. The incident light propagating through the lead-in SMF is split into two parts once it arrives at the front splicing joint. One propagates along the residual core of the DF (*I_DF_*), while the other propagates in the side-opened MC (*I_MC_*). At the rear spicing joint, the two beams interfere with each other and part of the interference light is then coupled into the guided core mode of the lead-out SMF, because of the mode-field mismatch between the DF and lead-out SMF. As a result, the lead-out interference light intensity *I_out_* could be described by the well-known dual-beam interference equation [20]:

(1) $$I_{out}\left(\lambda \right)=I_{MC}+I_{DF}+2\sqrt{I_{MC}I_{DF}}\cos\left(\varphi \right),$$

where *I_MC_* and *I_DF_* represent the light intensities of the beams propagating through the MC and DF, respectively, *φ* = 2*πL*Δ*n_eff_*/*λ* refers to the phase difference between the two beams, Δ*n_eff_* = *n_DF,eff_* − *n_MC,eff_* is the effective RI difference between the DF *n_DF,eff_* and side-opened MC *n_MC,eff_*, *L* represents the MC length, and *λ* is the free space wavelength in vacuum.

According to Equation (1), when the phase difference *φ* = (2*m* + 1)*π*, here *m* is an integer, the transmission presents minima. The corresponding central wavelength of the *m*th order interference dip *λ_m_* could be described as *λ_m_* = 2*L*Δ*n_eff_*/(2*m* + 1). The central wavelength difference between two adjacent interference dips (free spectral range, FSR) could be expressed as:

(2) $$FSR=\frac{\lambda_{m}\lambda_{m-1}}{L\Delta n_{eff}},$$

It can be observed that the FSR of the proposed MCMZI sensor is inversely proportional to the MC length and the effective RI difference between the two interference optical paths, which is verified from the subsequent experimental results.

Figure 1b–e illustrate the fusion splicing procedure for the proposed MCMZI device fabrication. The D-shaped fibers used in the splicing process were prepared beforehand by polishing a half section of the coating-removed standard SMFs (Corning SMF-28e) using a wheel-based side polishing technique [21,22,23]. The polish region length is 10 mm and the residual fiber thickness of the flat section is around half of the cladding diameter, ~62.5 μm with a deviation around 1 μm. In the later stages of polishing process, a finer polish was performed to make the side-polished surface smoother so as to reduce the light scattering loss. The fusion splicing process for the fabrication of fiber MCMZI sensor was completed using a commercial fusion splice machine (FITEL S178 A). Firstly, as shown in Figure 1b, the half-polished fiber with D-shaped cross-section was cleaved into two segments. Here, it is should be noted that the end face of DF must be cleaved flatly enough to ensure that the subsequent fusion splice with good quality. Secondly, a segment of the cleaved DF and a standard SMF were clamped into the fiber holders in the fusion splicer and, with manual operation mode, the DF was stepped approaching to the SMF, and calibrated downward or upward to make the residual core of DF just aligned with half core of the SMF in *x* direction while no lateral offset existence in *y* direction, as shown in Figure 1c. After completing this manual calibration, arc discharge was performed to splice the DF with the SMF. The splicing parameters are as follows: the discharge intensity is set to “70” and the duration time is set to 190 ms. The fusion splicing process is very important because it must be ensured that the interface of DF and SMF has no obvious deformation. During the splicing process, we purposely deviated the splicing point for a short distance from the central arc region, to prevent the DF from being damaged due to over intensive arc discharge. After splicing the front splicing joint, a section of DF with predesigned length was cleaved with associated assistances of a linear translation stage and a real-time CCD camera, as shown in Figure 1d. Finally, the other free end face of the DF was spliced with another SMF with the same relative calibration as the front splicing joint to form the rear splicing joint, as shown in Figure 1e. In the operation process, the transmission spectrum was also simultaneously monitored to get the best calibration. In this way, two SMFs were spliced into a segment of DF, with the length from tens to hundreds of micrometers, to construct a side-opened MCMZI, as shown in Figure 1a. The in-fiber side-opened MC allows reversible replacement of various liquids with different RIs.

**Figure 2:**
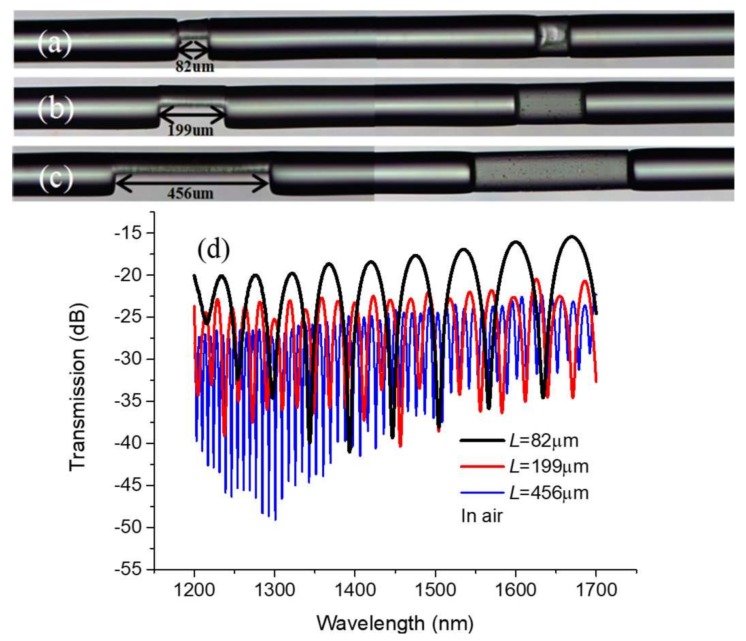
(**a**–**c**) Side and top view microscopic images of the MCMZIs with different cavity lengths of 82 μm, 199 μm, and 456 μm; (**d**) Transmission spectra of the MCMZIs in air corresponding to (**a**–**c**).

By repeating the above procedures, three DF-based MCMZIs with different MC lengths of 82 μm, 199 μm, and 456 μm were fabricated. The optical microscope images are shown in Figure 2a–c respectively. Considering the light in the reference arm mainly propagates along the residual core layer of the DF after polishing, the slight defect in the fiber cladding layer does not seriously affect the device performances. Figure 2d shows the experimentally measured transmission spectra in air of the above three devices, illustrated in Figure 2a–c. It could be seen that the three transmission spectra exhibit typical dual-beam interference with the fringe visibility of 15~20 dB, which is easily sufficient for most wavelength-interrogated sensing measurements. Due to the light scattering loss, the MC with longer cavity length would experience a slightly larger transmission loss. It is obvious that the FSR of the three transmission spectra decreases with the increment of cavity length, which is in agreement with the FSR definition formula expressed by Equation (2).

## 3. Principle Simulation and Experimental Setup

### 3.1. Simulation Results and Analysis

**Figure 3:**
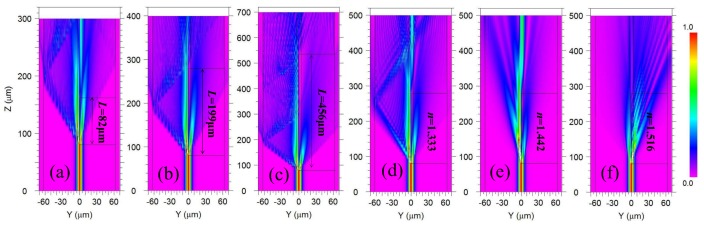
Simulated electric field intensity evolution for light propagating through the MCMZIs when the cavity length is (**a**) 82 μm; (**b**) 199 μm; and (**c**) 456 μm in air, and when ambient RI is (**d**) 1.333; (**e**) 1.442; and (**f**) 1.516 for the cavity length 199 μm.

To better understand the interference mechanism of our proposed structure, the commercial BeamPROP module (Rsoft Inc., Folsom, CA, USA), based on the beam propagation theory, was employed to simulate the electric field intensity evolution process as the incident light propagates through the above three MCMZIs, with different cavity lengths in air and under different ambient RI environments, as illustrated in Figure 3a–f. The black outlines in the figures illustrate the contours of the fiber cladding, fiber core, and MC regions. From Figure 3a–c, it can be seen that the incident light mainly separates into two portions at the front splitter joint. These two portions of light propagate through the MC and the residual core layer of the DF respectively, and thus experience difference optical paths; their spectral interference occurs at the rear combiner joint. Due to the scattering, the lead-out interference light for the structure with longer cavity length would experience a larger transmission loss. Comparing cases of different ambient RIs for the same cavity length of 199 μm, as shown in Figure 3b,d–f, it can be seen that more and more light energy leaks into the opening cavity with the increase of RI. When the ambient RI increases to 1.516, the light energy in the MC exceeds that in the DF as shown in Figure 3f.

### 3.2. Experimental Setup

**Figure 4:**
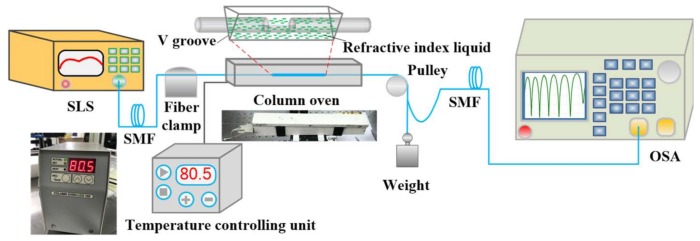
Schematic diagram of the experimental setup for the temperature measurement system. Insets, the physical photographs of the two separated parts of temperature oven: a column oven and the temperature controlling unit.

The temperature sensitivity of the proposed MCMZI sensor is obtained by wavelength interrogation of MZI transmission spectra. Figure 4 shows the schematic experimental setup for the temperature measurement system. The transmission spectra are measured by employing a super-continuum light source (SLS, Fianium Whitelase Micro, Southampton, UK), in order to provide broadband spectral output from 1200 nm to 1700 nm, and by utilizing an optical spectrum analyzer (OSA, Yokogawa AQ6370, Tokyo, Japan) with a wavelength resolution of 0.02 nm to monitor the transmission spectrum in real time. During the measurement process, the MCMZI sensor is placed horizontally in a temperature controlled oven (LCO 102 manufactured by ECOM, Praha, Czech Republic) with a temperature resolution of 0.1 °C, which is employed to ensure a stable environment temperature ranging from ambient temperature up to 99 °C. The temperature oven mainly includes two separate parts: a column oven and a temperature-controlling unit, as the physical photographs shown in Figure 4 insets. A copper V-groove is placed inside the column oven as the RI liquid carrier used to immerse the MCMZI sensor. During the whole experimental process, the in-fiber sensor passes through the two apertures at the two ends of the lidded column oven, and is fixed by a fiber clamp at one end; the other end is applied using an axial force by loading a weight (10 g) through a fixed pulley to keep the fabricated structure stretched straight and without stress perturbation, as shown in Figure 4. In the experiment, various standard index-matched liquids (Cargille Labs, Cedar Grove, NJ, USA) with different RIs were used to fill the MC, for investigating the temperature response of transmission spectral characteristics of the proposed MCMZI sensor. Each time that a liquid sample was measured, the MCMZI device was subsequently cleaned using alcohol and a distilled water droplet hung on the tip of a pipette, until the transmission spectrum restored to the original state in air, as shown in Figure 2d.

## 4. Sensing Experiments and Discussion

### 4.1. Spectral Response to Ambient Temperature

**Figure 5:**
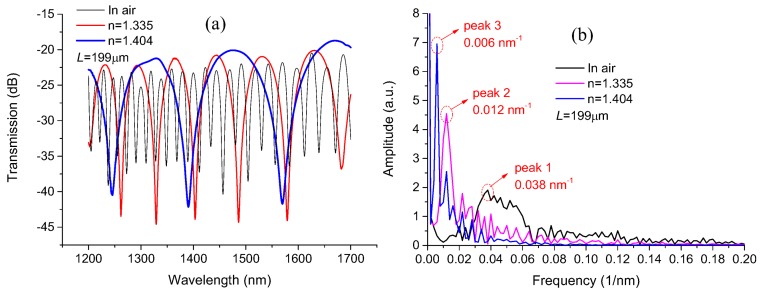
(**a**) Transmission spectra and (**b**) spatial frequency spectra of the MCMZI in air and different RI liquid environments, for the device with cavity length 199 μm.

By controlling the environmental temperature, the RIs of the silica-based fiber and the various liquids filled in the MC would change in different degrees because of their different TOCs; meanwhile, the MZ interference spectra will shift accordingly. By monitoring the spectral evolution, it is convenient to carry out temperature measurements via the proposed MCMZI sensing system. Figure 5a shows the transmission spectra of the fabricated MCMZI sensor, with cavity length 199 μm immersed in air and in two different liquids of *n* = 1.335, and 1.404 environments. Apart from being related to the cavity length *L*, the transmission spectrum of the proposed MCMZI structure is intimately linked to the RI *n* of the liquids filled in the MC. From Figure 5a, it can be seen that the spectral FSR increase with the increase of the RI in MC, which is in accordance with the FSR definition formula, Equation (2). Compared to the case in an air environment, the interference spectral fringe visibilities of the devices with the filled liquid RIs of 1.335 and 1.404 were enhanced, due to the reduction of light intensity difference in the two interference arms. Here, it should be noted that these variation regularitiesare based on the premise that the RI of cavity has not exceeded that of the reference arm.

The spatial frequency spectra can reveal the number and amplitude distribution of the modes participating in the modal interference process. By performing a Fourier transform on the transmission spectra shown in Figure 5a, the corresponding spatial frequency spectra in Figure 5b could be obtained with the spatial frequency described as *ν =* Δ*n_eff_L/λ*^2^ [24]. Different peaks in the spatial frequency spectrum normally correspond to the modal interference between fundamental modes and different high-order modes. From Figure 5b, it can be found that there is more than one peak around the main peak (Peak 1, ~0.038 1/nm) in the spatial frequency spectrum for the air environment, which implies that several higher modes dominantly participate in the modal interference process in this case. However, when the MC is filled with index-matched liquids of RI 1.335 and 1.404, two dominant peaks specified as Peak 2 (~0.012 1/nm) and Peak 3 (~0.006 1/nm) respectively emerge in their spatial frequency spectra, which implies more light energy leaks into the opening cavity and fewer modes participate in the modal interference process. Moreover, comparing the frequency positions where these three dominant peaks were located at, it is apparent that high spatial frequency components are suppressed; the dominant frequency peak moves toward a lower frequency region with the increase of the filled liquid RI. This is due to a certain amount of reduction in the effective RI difference between the DF and MC arms.

For MZI sensors, monitoring an interference dip wavelength *λ_m_* shift in the transmission spectrum in response to the measure and variation is a commonly employed interrogation approach. When the in-fiber MCMZI sensor is immersed in a fluid, attributing to the different TOCs of the fluid and the silica-based fiber, the effective RI difference of the two interference arms will change accordingly in response to the environment temperature variation, which would lead to the temperature-dependent spectral dips shift. In addition, the thermal expansion of the silica-based fiber as the reference arm also should be considered. Hence, the temperature sensitivity of a certain spectral dip for the MCMZI sensor can be described as [25]:

(3) $$S_{Temp}=\frac{d\lambda_{m}}{dT}=\lambda_{m}\left(\frac{1}{\Delta n_{eff}}\frac{d\Delta n_{eff}}{dT}+\alpha \right),$$

where, *α* = 4.1 × 10^−7^/°C is the thermal expansion coefficient of the silica-based fiber [26]. 

**Figure 6:**
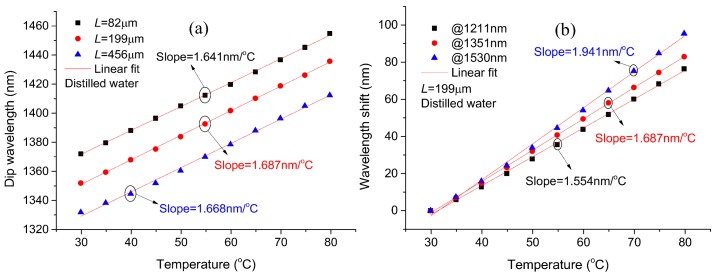
(**a**) Spectral dip wavelength as functions of environmental temperature for the proposed device with different cavity lengths of 82 μm, 199 μm, and 456 μm; (**b**) Spectral dip wavelength shift as functions of environmental temperature at different dip wavelength positions for the device with cavity length 199 μm.

From the definition formula of temperature sensitivity *S_Temp_*, Equation (3), it is clear that the sensitivity is irrelevant to the cavity length, and that a longer dip wavelength will mean higher sensitivity. In order to prove these realities through experimentation, the devices were measured in distilled water under a temperature range of 30 °C to 80 °C, as shown in Figure 6. Figure 6a gives the experiment data and the corresponding fitted relationship lines of the dip wavelengths versus the environmental temperature for the proposed MCMZIs with three different cavity lengths. The slope of the fitting linear represents the corresponding temperature sensitivity. As illustrated in Figure 6a, the sensitivities for the devices with cavity lengths of 82 μm, 199 μm, and 456 μm in distilled water are 1.641 nm/°C, 1.687 nm/°C, and 1.668 nm/°C, respectively. From these experiment data linear fitting results, it can be seen that the temperature sensitivities are nearly equal for devices with different cavity lengths, which verifies the sensitivity independence from the cavity length. Figure 6b depicts the relationships of the interference dip wavelength shift versus the environmental temperature for the device with cavity length 199 μm, at a different starting dip wavelength. It illustrates that the temperature sensitivities are 1.554 nm/°C, 1.687 nm/°C, and 1.941 nm/°C at dip wavelengths 1211 nm, 1351 nm, and 1530 nm, respectively. Obviously, the longer the dip wavelength is, the higher the corresponding temperature sensitivity is, which is in accordance with Equation (3). Simultaneously, it should be noted that the dip wavelength only exerts a tiny influence on sensitivity.

**Figure 7:**
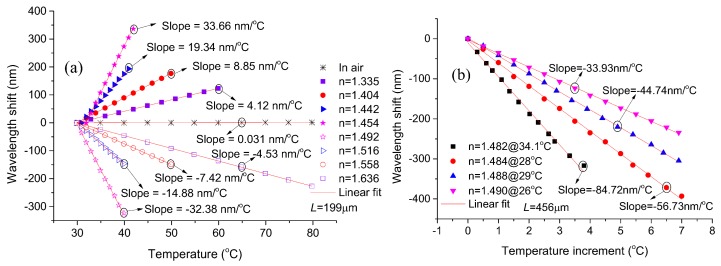
Spectral dip wavelength shift as functions of environmental temperature for the proposed device with cavity lengths (**a**) 199 μm and (**b**) 456 μm at different RIs of the filled liquids.

To demonstrate the temperature sensing performance of the proposed MCMZI sensor under different RI environments, the linear fitting for temperature responses of the device with cavity length of 199 μm placed in air (RI = 1.0002635, TOC = −7.592 × 10^−7^/°C) [27] and in various RI-matched liquids are depicted in Figure 7a. As Figure 7a shows, when the filled liquid has an RI of no more than 1.454, the transmission dips of this device exhibit red-shift in response to the increment in temperature, and the temperature sensitivity enhances with the increase of the liquid RI. However, as the liquid RI exceeds 1.492, the interference dips convert to blue shift and the sensitivity starts to reduce with the temperature increase. These phenomena result from the effective RI difference of the two interference arms, and from the fact that the filled liquid TOCs are larger than silica-based fiber. That is to say, as the effective RI of the MC arm is less than that of the DF arm, the RI difference between the two interference arms enhances with the increment of temperature due to the large TOC of the liquid filled in the cavity, which results in red shift of the interference dips. When the RI of cavity-filled liquid is so large that the effective RI of the DF arm is less than that of the MC arm, the RI difference of the two interference arms reduces with the increment of temperature. In this case, the interference dips tend to blue shift in response to the increment of temperature. Between the conversion of the red-shift and the blue-shift, there is a turning point corresponding to the nearly equivalent effective RI in the two interference arms. As the effective RIs of the two interference arms tend to equal, the sensitivity and FSR would sharply increase, which can be demonstrated using Equations (2) and (3) respectively.

In the ultrahigh sensitivity and large FSR region, the spectral range (see Figure 5a) imposes a restriction on the measurement range for the temperature sensing. Too large an FSR would lead to an interference dip shift which is out the OSA monitor spectrum range. A long cavity length can reduce the FSR, thus mitigating the monitor spectrum range limitation. In Figure 7b, the proposed MCMZI with cavity length 456 μm was employed to investigate the temperature sensing performance with higher sensitivity. As the linear fitting results for experiment data shown in Figure 7b, the MCMZI filled with the liquid of RI 1.482 possesses an ultrahigh temperature sensitivity of −84.72 nm/°C, approximately as 2733 times higher than that of the device in air (0.031 nm/°C). The dramatic enhancement of temperature sensitivity could be attributed to two aspects: one is that the TOCs of the RI-matched liquids (−0.000395/°C) is much larger than that of the silica-based fiber (silica: ~7.97 × 10^−6^/°C [28]), and the other lies in the proximity of effective RIs in the two MZ interference arms.

From the experimental results in Figure 7a,b, it can be seen that a wide range of temperature sensitivities from 33.66 nm/°C to −84.72 nm/°C may be achieved by filling the side-opened cavity with the corresponding RI liquids. The high sensitivity is inevitably limitedby the small sensing range. According to the specific requirements for temperature sensing applications, the sensor device having an appropriate sensitivity and measurement range can be fabricated by selectively sealing the corresponding RI liquid-infiltrated MCMZI in a microfluid or a capillary [8].

### 4.2. Ultrasensitive Region Analysis

**Figure 8:**
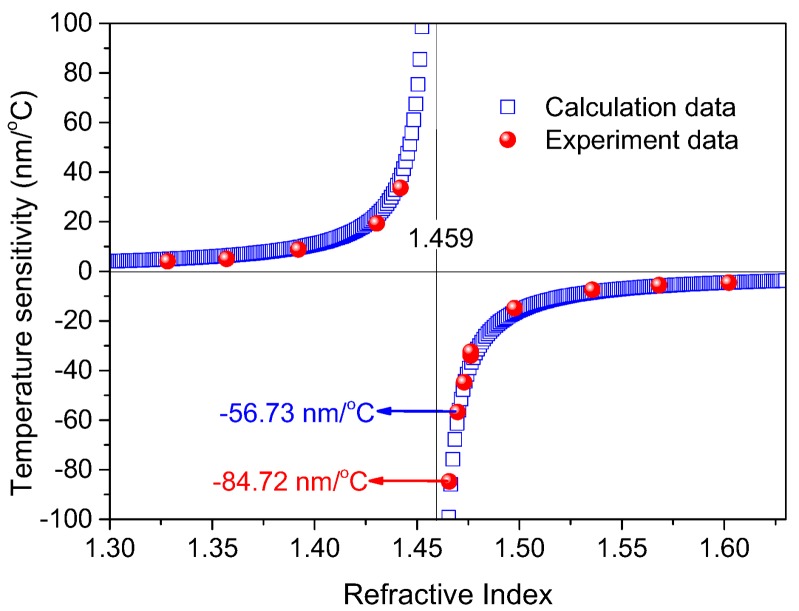
Comparison of theoretically calculated and experimentally acquired temperature sensitivities for the filled liquids with different RIs.

A series of RI-matched liquids covering an ultra-wide RI range from 1.335 to 1.636 have been used to experimentally measure the temperature sensitivities at different ambient RI in MC for the proposed MCMZI sensor, as shown in Figure 8. In the figure, the theoretical calculation data of temperature sensitivities were obtained from Equation (3) by assuming *λ_m_*= 1600 nm, *n* = 1.459, TOCs of D-shaped fiber and RI-matched liquid *dn_DF_*/*dT* = 7.97 × 10^−6^/°C and *dn_Lq_/dT* = −0.000395/°C, respectively. It can be seen that the experimentally acquired data for temperature sensitivity is in good agreement with the theoretically calculated data. The ultrahigh temperature sensitivities −56.73 nm/°C and −84.72 nm/°C were experimentally achieved. Moreover, the ultrasensitive region locates in the region of RI 1.459. Before the RI exceeds 1.459, the temperature sensitivity increases with the increase of RI, whereas after that, the temperature sensitivity decreases with the increase of RI.

**Figure 9:**
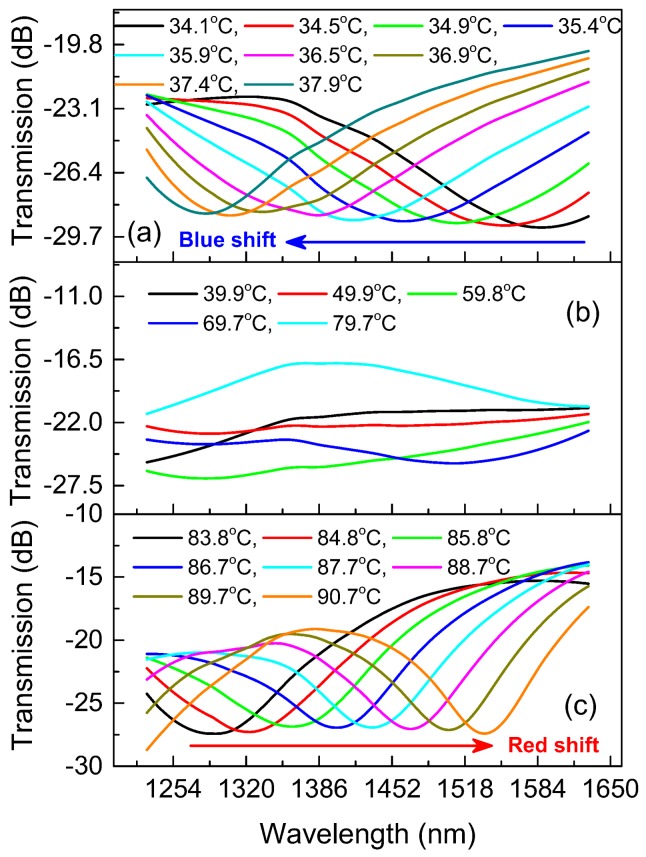
The transmission spectra evolution with the increase of environmental temperature, (**a**) 34.1 °C~37.9 °C, (**b**) 39.9 °C~79.7 °C, and (**c**) 83.8 °C~90.7 °C, for the device with cavity length of 456 μm and filled liquid RI of 1.482.

By increasing the temperature from 34.1 °C to 90.7 °C, the conversion process of the transmission spectrum dips from blue shift to red shift was experimentally observed in one device with cavity length of 456 μm and filled liquid RI of 1.482, as shown in Figure 9. When the temperature increases from 34.1 °C to 37.9 °C, the spectrum dips blue shift with the increase of temperature, as shown in Figure 9a. This is because that the effective RI of the cavity is larger than that of the DF, whereas the RI difference reduces with the temperature increased due to negative TOC of the filled liquid. As shown in Figure 9b, the interference dips shift out of the spectral range monitored by the OSA in the temperature range of 39.9 °C to 79.7 °C, which results from that the reduction of effective RI difference of the two interference arms, leading to a in sharp increase in the FSR. As the temperature increases from 83.8 °C to 90.7 °C, the spectrum dips happen to be red shift increases in temperature, as shown in Figure 9c. The reason is that the effective RI of the cavity is less than that of DF, whereas the increase of temperature enhances the effective RI difference.

### 4.3. Comparison and Discussion

Table 1 exhibits sensing performances of various temperature sensors based on different structures and fabrication methods. For the reported temperature sensors based on various diffraction or interference structures of optical fiber itself, such as LPFGs [6], FPI based on hollow-core rod (HCR) [5], MI based on peanut-shape structure fiber (PSSF) [7], and MZI based on waist-enlarged bitapers (WEB) [16], temperature sensitivities are limited to several or tens of picometers per centigrade, due to the small TOCs of the optical fibers based on silica material. Moreover, some fiber-optic interferometers were fabricated based on the interference between fiber cladding modes and the core mode with small effective RI difference, which results in whose sensing area length for accumulating enough phase difference are a few even tens of millimeters long [7,16]. Drawing support from the large RI difference between of fiber core and air cavity, a MZI sensor based on an air MC fabricated by femtosecond laser micromachining reduced the sensing area length of the device to 47 μm [2]. However, the obtained temperature sensitivity is only ~0.046 nm/°C, which results from the large effective RI difference between two MZ interference arms and the small material TOCs. Some reported works filled high TOC liquid into the PCF for temperature sensing by use of femtosecond laser-assisted selective infiltration method to improve temperature sensitivity [17,19]. Although, the sensitivities were dramatically enhanced to tens of nanometers per centigrade, these devices need complicated fabrication technologies and have sensing area of a few centimeters long [16]. As experimentally demonstrated in this study, the temperature sensitivity −84.72 nm/°C of the proposed MCMZI sensor is the highest amongst the reported fiber-based temperature sensors, which exceeds the sensitivity 16.49 nm/°C of the extremely high-sensitive liquid-filled PCF sensor [18] by a factor of 5.14. Simultaneously, the sensing area length of our sensor is reduced to hundreds of micrometers from several centimeters. Moreover, the fabrication process for the device is not complicated, involving only fiber polishing and fusion splicing.

## 5. Conclusions

In conclusion, we have proposed and experimentally demonstrated a miniature inline optical fiber MCMZI sensor based on a liquid-filled D-shaped fiber cavity for temperature measurement with ultrahigh sensitivity. The in-fiber MCMZI sensor was constructed with a section of DF fusion spliced between two SMFs to form a side-opened MC with cavity size in the range of tens to hundreds of micrometers. Combining the favorable characteristics of the MCMZI structure and the high TOC liquid filled into the side-opened cavity, this device exhibits outstanding temperature sensing performance. A wide range of temperature sensitivities from 33.66 nm/°C to −84.72 nm/°C have been experimentally achieved by filling different RI liquids into the MC. The ultrahigh temperature sensitivity (up to −84.72 nm/°C in the temperature range of 34.1 °C~37.9 °C) for a device with cavity length of 456 μm is the highest temperature sensing sensitivity for the reported fiber temperature sensors, to the best of our knowledge. Experimental and calculated results are in good accordance with each other and simultaneously indicate that the interference spectra show extreme temperature sensitivity, as effective RI of the MC approaching to 1.459. Moreover, the conversion process of interference dips from blue shift to red shift with the temperature increasing from 34.1 °C to 90.7 °C was experimentally observed in one device. Therefore, such an MCMZI device could be used to develop a promising temperature sensor due to its outstanding advantages of selectable ultrahigh sensitivity, simple configuration, and miniature scale.

## Figures and Tables

**Table 1:** Comparison of various fiber temperature sensors based on different structures and fabrication methods.

Sensor Structure	Fabrication Technique	Sensing Area Length	Temperature Range (°C)	Sensitivity (nm/°C)	Liquid RI	TOC (/°C)
LPFG-filter [6]	fs laser direct writing	5 mm	20~500	−0.01552	-	-
DPM-FLM [4]	mechanical polishing	10 mm	30~80	0.13	-	-
PSSF-MI [7]	Special fusion splicing	21 mm	100~900	0.096	-	-
WEB-MZI [16]	Special fusion splicing	8 mm	30~1000	0.087	-	-
PCF-MMI [17]	fs laser micromachining	2.2 cm	18~21	14.72	1.48	−3.95 × 10^−4^
PCF- MZI [18]	Direct manual gluing	2.5 cm	20~25	16.49	1.454	−3.90 × 10^−4^
MCMZI (This work)	Special fusion splicing	456 μm	28~35	−56.73	1.484	−3.96 × 10^−4^
			34.1~37.9	−84.72	1.482	−3.95 × 10^−4^
